# 肺癌患者胸腔镜术后主要症状变化规律分析

**DOI:** 10.3779/j.issn.1009-3419.2022.101.23

**Published:** 2022-06-20

**Authors:** 曦 陈, 映显 董, 娇 王, 彦 王, 钧科 常, 凤 陈, 梅 杨, 国卫 车

**Affiliations:** 1 610041 成都，四川大学华西医院患者全程管理中心/四川大学华西护理学院 Department of Integrated Care Management Center, West China Hospital, Sichuan University/West China School of Nursing, Sichuan University, Chengdu 610041, China; 2 610041 成都，四川大学华西医院患者全程管理中心/四川大学华西胸外科 Department of Thoracic Surgery, West China Hospital, Sichuan University/West China School of Nursing, Sichuan University, Chengdu 610041, China; 3 610041 成都，四川大学华西医院患者全程管理中心/四川大学华西康复科 Department of Rehabilitation, West China Hospital, Sichuan University/West China School of Nursing, Sichuan University, Chengdu 610041, China

**Keywords:** 胸腔镜手术, 疼痛, 咳嗽, 肺肿瘤, Video-assisted thoracoscopic surgery, Pain, Cough, Lung neoplasms

## Abstract

**背景与目的:**

肺结节患者通过微创手术进行治疗，术后相关症状成为影响患者情绪和生活质量的主要因素，本研究旨在分析肺结节肺癌患者术后症状变化的规律。

**方法:**

前瞻性分析四川大学华西医院胸外科2021年6月-2021年9月单个医疗组88例肺癌患者的临床资料。分析术前、出院当天、出院30天及90天的临床症状种类及其严重程度。

**结果:**

肺癌患者术后症状发生率79.5%，且以轻度(54.3%)、中度(32.9%)症状为主。肺癌患者术后主要症状有疼痛(55.7%)和咳嗽(37.2%)。疼痛发生率在出院时(55.7%)均显著高于出院30天(23.7%)和出院90天(12.0%)(*P*=0.01, *P*=0.01)。咳嗽发生率在出院30天(66.1%)和90天(66.0%)均显著高于出院时(37.2%) (*P*=0.01, *P*=0.04)。

**结论:**

肺结节肺癌患者术后主要症状是疼痛和咳嗽，且疼痛发生率和程度均随时间而减少或减轻，而咳嗽发生率随时间增高但程度逐渐减轻。

## Abstract (en)

**Background and objective:**

Patients with pulmonary nodules are treated by minimally invasive surgery, and postoperative symptoms have become the main factors affecting patients' emotion and quality of life. This study aimed to analyze the changes of postoperative symptoms in lung cancer patients with pulmonary nodules.

**Methods:**

The clinical data of eighty-eight lung cancer patients admitted to the same medical group of Department of Thoracic Surgery, West China Hospital of Sichuan University from June 2021 to September 2021 were prospectively collected and analyzed. The types and severity of clinical symptoms before operation, on discharge day, 30-day and 90-day after operation were analyzed.

**Results:**

The incidence of postoperative symptoms in lung cancer patients was 79.5%, and most patients suffered from mild (54.3%) and moderate (32.9%) symptoms. The main postoperative symptoms of lung cancer patients were pain (55.7%) and cough (37.2%). The incidence of pain at discharge (55.7%) was significantly higher than that at 30-day (23.7%, *P*=0.01) and 90-day (12.0%, *P*=0.01) after discharge. The incidence of cough was significantly higher at 30-day (66.1%) and 90-day (66.0%) than that at discharge (37.2%) (*P*=0.01, *P*=0.04).

**Conclusion:**

The main postoperative symptoms of lung cancer patients with pulmonary nodules are pain and cough. The incidence and severity of pain decreases with time, and the incidence of cough increases but the severity decreased gradually.

随着健康人群体检意识的增高和胸部低剂量螺旋计算机断层扫描(computed tomography, CT)的广泛应用，肺结节诊断为肺癌的患者通过应用胸腔镜进行手术，既降低了围手术期并发症，又改善了临床结局^[1, 2]^。但是手术本身也会为患者带来一些临床症状，研究^[3, 4]^表明肺癌患者术后的主要症状是疼痛、咳嗽和疲劳，这些症状至少可以持续3个月或更长，是导致患者焦虑烦燥的主要因素并影响其生活质量。目前微创手术患者的住院时间显著缩短，甚至可以进行日间手术，这些症状的治疗和改善需要社区或家庭康复^[5-7]^。问题是目前关于肺癌患者术后症状变化规律及治疗方案的研究尚未见报道。我们前瞻性地分析了肺癌患者术前、出院当天、出院30天及90天患者的临床症状，初步探讨了这些症状的严重程度及变化规律，以便为后续治疗提供指导方案。

## 1 资料与方法

### 1.1 临床资料

连续分析2021年6月-2021年9月在四川大学华西医院胸外科行胸腔镜肺癌肺叶或肺段切除术的患者110例。纳入标准：①病理学检查诊断为原发性肺癌；②手术方式为电视辅助胸腔镜手术(video-assisted thoracic surgery, VATS)肺叶(肺段、单叶或双叶)+系统淋巴结清扫术；③术前无严重疼痛、慢性阻塞性肺疾病、哮喘和咳嗽病史；④临床资料完整。排除标准：①临床资料不完整；②开放手术的肺癌患者或全肺切除患者；③术后出血或持续漏气需要再次手术的患者；④术前行新辅助治疗患者。110例患者中，其中良性17例，资料不完整者5例，最终纳入肺癌患者88例，平均年龄(48.95±10.50)岁；男性28例，平均年龄(51.18±14.13)岁，女性60例，平均年龄(44.27±18.21)岁；体重指数(body mass index, BMI)(22.64±3.26)kg/m^2^；吸烟患者16例(18.2%, 16/88)；其中合并高血压患者16例(18.2%, 16/88)，糖尿病患者5例(5.7%, 5/88)，结核患者1例(1.1%, 1/88)，胸部手术史患者3例(3.3%, 3/88)；第一秒用力呼气量(forced expiratory volume in one second, FEV_1_)平均值(2.87±1.33)L/s；平均手术时间(67.18±20.3)min；腺癌88例，均为Ⅰ期，术后分期采用国际抗癌联盟(Union for International Cancer Control, UICC)(2018)肺癌分期标准。平均住院日(5.01±1.96)d，术后平均住院日(2.99±1.33)d。所有患者均无明显需要临床治疗的并发症。

### 1.2 方法

#### 1.2.1 手术方式

单向式胸腔镜解剖性肺切除法+系统(或特异性)淋巴结清扫^[8]^。肺叶、段间平面用切割缝合器处理。淋巴结清扫，纵隔或肺内淋巴结不少于3组，且第7组淋巴结必须清扫(左侧淋巴结第4、5、6、7、8、9、10、11、12组，右侧淋巴结第2、4、7、8、9、10、11、12组^[9]^)。

#### 1.2.2 管道管理

##### 1.2.2.1 尿管管理

所有患者术中及术后均不留置尿管^[10]^。

##### 1.2.2.2 胸腔引流管^[11]^

所有患者均用18 F硅胶球囊导尿管。术侧第3或4肋间置入，球囊需注入15 mL生理盐水，接水封引流瓶。引流管正常管理，术后24 h，患者咳嗽时若无漏气，且引流液颜色及量均正常，拍片肺复张，且胸腔无积液和积气，拔除引流管。

#### 1.2.3 镇痛方法^[7]^

患者围手术期均采用区域肋间神经阻滞麻醉镇痛，术后若必要时加用非甾体抗炎药(nonsteroidal antiinflammatory drugs, NSAID)(如帕瑞昔布或氟比洛芬酯注射液，按说明书用)，或口服布洛芬缓释胶囊或氨酚羟考酮片(按说明书用)。

#### 1.2.4 术后饮食^[12]^

患者术后均应用短期中链甘油三酯(medium-chain triglycerides, MCT)食谱；术后4 h，神志清楚后口服100 mL温开水，6 h-8 h饮用开胃流质250 mL，术后10 h-12 h口服50 g营养粉，兑温水250 mL，术后第1天，营养科订餐，MCT饮食，可喝水，进食水果。

### 1.3 观察指标

#### 1.3.1 术后并发症^[13]^

包括：(1)腹泻；(2)过敏反应；(3)皮下气肿；(4)心律失常；(5)小便失禁；(6)术后胸腔积气：胸部X线片提示：胸腔积气 > 30%；(7)术后胸腔积液：胸部X线片提示：胸腔积液中度以上；(8)肺部感染^[8]^：①明确的病原学证据；②影像学提示肺不张或大片状影；③发热；④白细胞总数大于10, 000/mL或15, 000/mL。

#### 1.3.2 症状及严重程度评估

应用肺癌患者术后症状量表进行评估^[14]^。

### 1.4 统计学分析

统计分析采用SPSS 16.0软件包，计数资料采用实际例数及百分比表示。计数资料比较采用卡方检验。*P* < 0.05为差异有统计学意义。

### 1.5 伦理审查

此研究已获四川大学华西医院生物医学伦理委员会批准(伦理批准号：2019-1115)。

## 2 结果

### 2.1 肺癌患者临床症状总体发生率及严重程度分析

74例(84.1%)肺癌患者术前无相关症状，有症状患者14例(15.9%)，其中轻度10例(13.5%)，中度4例(4.5%)。肺癌患者术后症状发生率在出院90天(56.8%)和出院后30天(67.0%)均显著低于出院时(79.5%)(*P*=0.01, *P*=0.03)；轻度症状发生率在出院90天(90.0%)均显著高于出院30天(67.8%)和出院时(54.3%)(*P*=0.02, *P*=0.01)。中度症状发生率在出院90天(8.0%)均显著低于出院30天(25.4%)和出院时(32.9%)(*P*=0.03, *P*=0.02)。见表 1。

**表 1:** 肺癌患者术后临床症状分析 Clinical symptom analysis of patients with lung cancer after operation

Item		AD (*n*=88)	DD (*n*=88)	DD30 (*n*=88)	DD90 (*n*=88)	*P*
Symptom	No	84.1%（74/88）	20.5%（18/88）	33.0%（29/88）	43.2%（38/88）	0.01
	Yes	15.9%（14/88）	79.5%（70/88）	67.0%（59/88）	56.8%（50/88）	0.02
Symptom severity	Mild	71.4%（10/14）	54.3%（38/70）	67.8%（40/59）	90.0%（45/50）	0.01
	Moderate	28.6%（4/14）	32.9%（23/70）	25.4%（15/59）	8.0%（4/50）	0.03
AD: admisson Day; DD: discharge Day; DD30: 30 days after discharge; DD90: 90 days after discharge.						

### 2.2 肺癌患者术后主要临床症状发生率分析

肺癌患者症状发生率在出院90天(56.8%)时显著低于出院时(79.5%)(*P*=0.02)。咳嗽发生率在出院30天(66.1%)和90天(66.0%)均显著高于出院时(37.2%)(*P*=0.01, *P*=0.04)。疼痛发生率在出院时(55.7%)均显著高于出院30天(23.7%)和出院90天(12.0%)(*P*=0.01, *P*=0.01)。疲劳发生率在出院时(7.1%)和出院30天(10.2%)均低于出院90天(22.0%)，但无统计学差异(*P*=0.11, *P*=0.24)。见表 2。

**表 2:** 肺癌患者术后主要临床症状分析 The main clinical symptom analysis of patients with lung cancer after operation

Item		DD (*n*=70)	DD30 (*n*=59)	DD90 (*n*=50)	*P*
Symptom category	Cough	37.2%（26/70）	66.1%（39/59）	66.0%（33/50）	0.01
	Pain	55.7%（39/70）	23.7%（14/59）	12.0%（6/50）	0.02
	Fatigued	7.1%（5/70）	10.2%（6/59）	22.0%（11/50）	0.11

### 2.3 肺癌患者术后主要临床症状严重程度分析

肺癌患者术后重度临床症状在出院90天(2.0%)均显著低于出院时(11.4%)和出院30天(6.8%)(*P*=0.01, *P*=0.01)。中度临床症状在出院90天(8.0%)均显著低于出院时(32.9%)和出院30天(25.4%)(*P*=0.01, *P*=0.02)。轻度临床症状在出院90天(90.0%)均显著高于出院时(54.3%)和出院30天(67.8%)(*P*=0.01, *P*=0.04)，见表 3。

**表 3:** 肺癌患者术后主要临床症状严重程度分析 Clinical symptom severity analysis of 88 patients with lung cancer

Symptom severity	DD (*n*=70)	DD30 (*n*=59)	DD90 (*n*=50)	*P*
Mild	54.3%（38/70）	67.8%（40/59）	90.0%（45/50）	0.04
Moderate	32.9%（23/70）	25.4%（15/59）	8.0%（4/50）	0.02
Severe	11.4%（8/70）	6.8%（4/59）	2.0%（1/50）	0.01
Extreme	1.4%（1/70）	0%（0）	0%（0）	0.13

## 3 讨论

近年胸部低剂量螺旋CT在体检中的广泛应用，使大量肺结节肺癌患者被发现，这部分患者多数均行胸腔镜手术，结合加速康复外科(enhanced recovery after surgery, ERSA)理念的广泛应用，使肺癌患者围手术期并发症(尤其是肺部感染)显著降低，住院时间显著缩短^[15, 16]^。体检发现的肺结节患者不但年轻化，且术后病理分期多数均是早期(Ⅰ期)，其生存率显著增高，但是这部分患者会因术后出现的症状而加重对肿瘤的焦虑^[17, 18]^。阐明肺结节肺癌术后主要临床症状变化规律，并针对性进行预防性治疗或向患者讲清楚，有助于减少患者的焦虑，并改善术后的生活质量^[19]^。

肺结节肺癌患者多数是在体检时被发现的，也有部分是进行临床检查时(如咳嗽或发热时)行胸部CT检查时发现，这些症状多数情况下与结节无关^[4]^。本研究88例患者，术前只有14例有症状，且10例为轻度，1例咳嗽为中度(事实是术前进行了纤支镜检查，入院时有咳嗽)。因此，本研究也再次证实肺结节患者术前应该无明显临床症状。术前的焦虑与担心肺结节是肺癌有关。肺结节术后的症状主要是疼痛、咳嗽、疲劳、恶心呕吐等，尤其以疼痛和咳嗽为主，这与我们以前的研究一致^[3, 20-22]^。本研究发现，术后早期到出院时以疼痛为主(55.7%)，且以轻度(54.3%)、中度(32.9%)为主。出院后30天以咳嗽为主(66.1%)，轻度(67.8%)、中度(25.4%)为主。出院后90天，所有症状均以轻度(90%)为主。而疲劳的发生率似有上升趋势，从出院时的7.1%、30天后的10.2%到90天时的22%，不但无统计学差异，也多数为轻度，且不影响正常生活。这可能与疼痛和咳嗽的减轻或消失，使疲劳相对增高有关。

疼痛的变化规律是出院时发生率最高，也以轻度为主，出院后30天减轻，90天后可转为麻木或消失。咳嗽出院时发生率低，30天时发生率达到高峰且多为中度，90天后转为轻度或消失，分析原因可能与术后气道或肺损伤有关，同时可能还与迷走神经损伤有关。疲劳90天后达到高峰。这些研究结果提示，需重视围手术期疼痛(切口和引流管口)处理^[23]^，出院后早期的咳嗽管理及肺康复训练(缓解疲劳)^[24]^。研究^[25]^表明，术前及术后的肺康复训练有助于降低咳嗽和疲劳的发生率及严重程度，同时有助于生活质量的改善。

本研究结果数据来源于单一医疗机构和同一医疗组，且多为早期肺癌患者，研究结果需要通过多中心验证。但是也给我们一定提示，肺结节肺癌患者术后的症状主要影响因素是麻醉和手术过程，需要我们尽量减少因手术带来的创伤，同时对可能的问题进行及时有效地预防或治疗。
